# Challenges and solutions of medical residency: the example of Iran

**DOI:** 10.1186/s12913-024-11263-x

**Published:** 2024-07-27

**Authors:** Behrooz Rahimi, Ali Nemati, Behzad Tadayon, Mahmood Samadpour, Amin Biglarkhani

**Affiliations:** 1 Deputy of Management Development, Resources and Planning, Ministry of Health & Medical Education, Tehran, Iran; 2 Department of health Services Management, School of Health Management and Information Sciences, Iran University of Medical Sciences, Tehran, Iran

**Keywords:** Residency, Employment, Challenges, Barriers, Solutions

## Abstract

**Background:**

Medical residency constitutes a highly demanding and taxing phase in the professional journey of physicians. Factors such as low income, excessive workloads, and uncertainty regarding their career trajectory can contribute to diminished job satisfaction among residents. Neglecting this issue can have enduring negative effects on the quality and quantity of healthcare services provided. This research aims to explore the challenges encountered during medical residency and propose viable solutions.

**Methods:**

In this qualitative study conducted in 2023, interviews were employed to identify challenges, facilitators, barriers, and potential solutions associated with transitioning from residency to a job. In the qualitative section, a purposive selection process led to the inclusion of 26 interviewees, and for the Delphi method, 17 experts were purposefully chosen at three hierarchical levels: macro (Ministry of Health), intermediate (university), and executive (hospital). Qualitative data underwent analysis using a conceptual framework, while Delphi data were subjected to quantitative analysis.

**Results:**

The qualitative analysis revealed five general themes with 13 main categories and 70 sub-categories as challenges, two general themes as facilitators, and barriers to the transition from residency to a job. Additionally, eight main categories with 52 interventions were identified as solutions to overcome these barriers. In the Delphi stage, the number of proposed solutions was streamlined to 44 interventions. The most significant challenges identified in this study included high workload during residency, low income, career-related uncertainties, issues related to welfare services, and challenges in education and communication.

**Conclusion:**

The decline in residents’ willingness, coupled with the substantial work and financial pressures they face, poses a serious threat to the healthcare system, necessitating significant reforms. Transitioning from residency to a full-fledged job emerges as a potential avenue to address a substantial portion of the expressed needs. Implementing these reforms demands resolute determination and collaboration with sectors beyond the healthcare system, integrated into a comprehensive national healthcare plan that considers the country’s capabilities.

**Supplementary Information:**

The online version contains supplementary material available at 10.1186/s12913-024-11263-x.

## Background

The medical residency phase represents a pivotal juncture for physicians globally, marked by significant physical and mental demands [1]. Trainees navigate heightened responsibilities, taxing workloads, sleep deprivation, physical fatigue, and comparatively modest salaries throughout this critical period [2]. This phase assumes particular significance in the medical profession, as evidenced by alarming rates of physician suicide, bringing attention to the pervasive issue of burnout [3]. The impact of job burnout during residency extends beyond professional implications, manifesting in personal adversities such as substance abuse and suicidal ideation [4]. Furthermore, burnout correlates with increased medical errors, diminished empathy, and reduced compliance with standards and safety protocols among resident physicians [5–8].

Medical residency entails a highly demanding and stressful period for physicians, characterized by stressors like high workload, extended shifts, inadequate supervisory support, and limited autonomy [9]. Escalating economic pressures, performance expectations, and heavy workloads often leave resident physicians disillusioned with modern medical practice, resulting in higher levels of burnout and depression compared to the general population and subsequently, lower job satisfaction [10–12]. In the field of medicine, especially during residency, where duties are of paramount importance, and the training program is strenuous, cultivating interest and job satisfaction becomes even more critical. Statistics reveal that approximately 10% of U.S. residents change their specialization during training [13].

In the United States, nearly half of practicing physicians undergo career transitions, adapting to demanding occupational requirements that significantly impact their emotional and intellectual well-being, fostering a healthy work-life relationship [14, 15]. Switzerland has emerged as the preferred destination for German immigrant doctors due to factors like better education, a harmonious work-life balance, and higher remuneration [16]. In Japan, residents’ salaries were insufficient, compelling them to take on second jobs to make ends meet, resulting in exhaustion and an inability to focus on their training [17]. To address these challenges, the Japanese government reformed the PGME system in 2004, introducing a new mandatory PGME program. In December 2017, resident work hours regulations were implemented for the first time in Korea [18]. These regulations aim to safeguard residency rights, contribute to high-quality education, and enhance patient safety. However, their effects on patient safety and assistant training remain controversial [19]. In Korea, several reports have highlighted substandard work environments, including excessive workload, sleep deprivation, and emotional exhaustion [20].

Historically, extended hospital shifts have been a defining feature of medical residency programs, sparking ongoing debates surrounding regulations governing working conditions. Issues such as low income, heightened workloads, challenges associated with frequent specialty rotations, and the pervasive uncertainty about career trajectories collectively contribute to diminished job satisfaction among residents within these programs [21, 22]. Job satisfaction, serving as an indicator of an individual’s contentment with their profession, bears a close correlation with stress levels, intentions to leave the profession, and the quality of healthcare delivered [23, 24].

In recent years in Iran, there has been a notable increase in the level of dissatisfaction among some medical residents. Additionally, there has been a noticeable decrease in the motivation among some general practitioners to enroll in this course. This widespread dissatisfaction has raised significant concerns about the current conditions of the residency program and its future trajectory. Residencies play an indispensable role in meeting medical workforce requirements, particularly in low- and middle-income countries, where they serve as significant contributors to public sector healthcare. In this context, resident physicians’ shoulder substantial responsibility for delivering quality medical services to meet population needs. However, it is crucial to recognize that residency serves as an educational system designed to equip doctors for future roles as medical specialists. Achieving this educational goal necessitates the provision of appropriate training and conducive implementation conditions. The challenges faced by resident’s demand solutions grounded in evidence-based information, underscoring the need for a comprehensive study that explores various dimensions from the perspectives of stakeholders and decision-makers. The primary objective of this study is to gather evidence and insights to formulate effective solutions in this critical domain.

## Methods

In this 2023 study, our primary objective was to delve into the perspectives and insights of experts and stakeholders. The qualitative methodology employed was centered around in-depth interviews, meticulously designed to unravel the challenges and solutions within the healthcare system transition from residency.

### Study design and participants

We purposefully selected 26 participants, ensuring representation from diverse roles, including medical residents, with a majority boasting over 10 years of experience in the healthcare sector. Participants spanned across all three hierarchical levels: macro (Ministry of Health), intermediate (medical university), and executive (hospital). The selection process continued until data saturation was attained.

### Data collection process

Each face-to-face interview, lasting between 40 and 60 min, adhered to a carefully structured protocol. The interviews began with a comprehensive informed consent from all of the participants, underscoring our commitment to confidentiality and participants were assured that all information would be anonymized during analysis and reporting.

### Analysis approach

Post-interviews, we initiated a meticulous process of implementation and coding. This phase involved extracting and categorizing subtopics related in data extraction form to the identified challenges and potential solutions. The conceptual framework analysis method guided this process, ensuring a systematic approach to the interpretation of qualitative data. For the analysis of interview data, we used MaxQDA software, ensuring a robust and efficient coding and grouping process.

Integration with Delphi Study: Following the initial phase of in-depth interviews, the qualitative insights were transformed into actionable interventions through a two-round Delphi method. This approach is widely acknowledged for its effectiveness in achieving consensus on complex and multifaceted topics.

### Round one

Drawing from the proposed interventions in preceding steps, the information was shared with a panel of 17 experts. In this initial round, experts and stakeholders, meticulously chosen for their expertise in the healthcare system, received questionnaires derived from the qualitative interview findings. These questions were designed to capture their individual perspectives on the identified challenges and potential solutions.

### Round two

Following the analysis of the results from the initial Delphi round, the interventions were once again submitted to the experts for review. In this phase of consensus-building, participants, equipped with anonymized feedback from the first round, actively participated in the second round of the Delphi process. The structure of this subsequent round was designed to facilitate convergence and consensus-building.

The trustworthiness is ensured through several key elements. The study’s transparent design, focusing on qualitative methodology through in-depth interviews, establishes clear objectives and provides a structured research approach. Purposeful sampling, involving 26 participants from diverse healthcare roles, including medical residents and experienced experts, enhances the credibility of the findings by ensuring comprehensive representation and two final interviews were to ensure the saturation of the interviews. Rigorous data collection procedures, including face-to-face interviews with informed consent, underscore ethical standards and contribute to the reliability of the collected data. Integration with a two-round Delphi method adds validation to the findings by engaging experts in consensus-building, thus strengthening the proposed interventions’ robustness and applicability. This iterative consensus-building process, involving experts with healthcare system expertise, ensures that interventions are informed by empirical evidence and expert opinion, reinforcing the study’s trustworthiness.

## Results

The theme of challenges during the residency period encompassed 11 main categories and 48 sub-categories, as outlined in Table 1. Varied perspectives emerged during interviews regarding the nature of residency, with some participants viewing it as an educational period and others as a job. For example, (P4) stated, “In my opinion, residency is an educational period, not a job.” In contrast, (P16) expressed, “Residency is a job.” This divergence in perception about residency was highlighted throughout the interviews. A hospital manager (P4) firmly asserted, “In my opinion, residency is an educational period and cannot be considered as a job.” Conversely, a different perspective was voiced by an attendee (P16), who stated, “Residency is a job, and a resident has certain responsibilities and tasks, along with some training; it is essentially a job that involves receiving specific training.” Furthermore, a healthcare financing specialist (P2) added their viewpoint, noting that “residency adds value… residents work from morning till night, treating patients. Residency is a job.”

**Table 1:** Medical residency challenges

Theme	Subtheme	Items
Context Challenges	The Economic and Social Situation of the Country	Inflation and High Prices, Political Issues and Sanctions, Apathy and Discontent in Society
	Cultural Factors (Individual and Societal Expectations)	High Societal Expectations, Differences in Individuals’ Expectations from the System, and Comparisons with Other Countries
Challenges in the Education System	Planning Challenges	Lack of a comprehensive, residency program, Mismatch between society’s needs and education Inadequate number of students relative to the workload in certain fields or regions, Insufficient educational infrastructure, including beds and faculty, in proportion to the number of students, Limited patient diversity due to a high number of students, Unclear job status for individuals before the start of their education, The need for a reassessment of the duration of residency programs in some fields, Excessive reliance on diagnostic technologies
	Inappropriate Resident Recruitment Methods	Selection of unmotivated and uninterested individuals, low skill levels and abilities of some residents, admission of incompetent individuals to the program, entry into fields without proper knowledge
	Challenges in Residency Training	Faculty members’ lack of familiarity with modern teaching methods, Inappropriate selection of faculty members, Inconvenient teaching hours, Inadequate or inappropriate educational curricula, Self-completion of theses by residents, Unclear job descriptions for residents in different years of the program, Thesis requirement as a graduation condition, Inadequate supervision of attending physicians over residents, Professors using their discretion to assign punishments to residents as a disciplinary tool
	Weak Existing Laws and Guidelines	Failure to Implement Existing Guidelines, Lack of Setting Shift Limits
Main Job-Related Challenges	Shortage of Welfare Facilities	Lack of Insurance, Absence of Resident Accommodations, Lack of Liability Insurance
	High Workload	Heavy Patient Loads, Excessive Work, High Patient Volume, Residents Handling a High Volume of Service Delivery
	Income Challenges	Inadequate Income Relative to Workload, Working in Private Hospitals, Rising Cost of Living, Livelihood Challenges
	Communication Challenges	Lack of appropriate communication between attendings and residents, Inappropriate confrontations, Military-like confrontations, Development of unhealthy behavior, Absence of attendings, Hierarchical abuse by lower-year medical students, Pressure on residents due to attendings’ dissatisfaction with government sector income
	Identity and Role Characteristics	Predominance of Therapeutic Role, Lack of Share in Hospital Income, Limited Decision-Making Power of Residents
	Future Career Challenges	Low Income during Training Program, Uncertainty about Future Job Prospects, Progressive Taxation Scheme, Low Income during Training, Limited Job Market
	Low Tariffs and Payment Delays	Low Tariffs, Payment Delays, Lack of Tariff Growth, Inadequate Attendings’ Income
Consequential Challenges		Presence of unmotivated and disinterested individuals, Resignations, Suicides, Physical and mental exhaustion of current assistants, Development of unhealthy behavior, especially after completing residency, Mistreatment of residents, Underground economy, Migration, Production of doctors for other countries, Lack of interest in residency programs by, doctors and a continuous decrease in residency applicants, Underutilized capacity, Increase in medical errors, Reduction in the quantity and quality of services provided

The initial subtheme, “Context Factors (Socioeconomic Conditions, Individual and Societal Expectations),” emerged as a key contributor to the challenges encountered during residency. Several interviewees highlighted economic issues and inflation as significant hurdles. (P1) observed, “Since 2014, when the electricity tariff lagged behind, it hasn’t improved much. Every year it’s around 10 to 15%, while inflation is between thirty to forty%. So, we’re facing a severe regression.” Echoing this concern, (P12) emphasized financial challenges, stating, “In 2017, surgery used to cost 9,000 tomans, but after 5 years, when I left that system, it had risen to 13,000 and something. Compare its growth to the country’s inflation rate. This, coupled with insurance companies’ delays in payments, demotivates all residents. With the delays they impose, given the inflation rate, it practically means 10 to 20% less for you. Consider this in a country with an 8-month delay. How much was the price of a car 8 months ago, and what is it now? The value of money keeps decreasing. There’s also a hidden tax here.”

Another internal factor discussed as a challenge in the context of resident expectations is the psychological pressure it places on residents. Participant (P6) noted, “You are a resident, you are a physician; everyone sees you as a specialist doctor, but you can’t even afford to pay for your child’s education, not even in a public exemplary school, let alone a private one. It’s natural that a person gets frustrated.” In line with this sentiment, another interviewee (P4) underscored the importance of income, stating, “Income is essential, and I really emphasize it. If I don’t have an income, even though I’m not young anymore, I was a general physician, then a specialist assistant, the family pressures me to get married. Now that I’m married, where should I get the resources to provide for my life?”

Within the second thematic category, Challenges in the Education System (Challenges in Residency Program Planning, Inappropriate Resident Recruitment Methods, Challenges in Residency Training Implementation, Existing Laws and Guidelines), participants addressed several noteworthy topics:

One highlighted issue was the absence of comprehensive planning for the residency program. Participant (P9) raised concerns, stating, “In the past, when society needed doctors, the average university admission rate was suitable for these individuals. Currently, the demand for doctors has decreased, but we are still admitting medical students, and the future prospects are uncertain.” A hospital resident (P10) conveyed, “They lack motivation. It’s disheartening when a graduate says they don’t know what to do.” Adding to this perspective, participant (P8) mentioned, “We don’t have any well-defined and comprehensive program at the macro level for the residency program.”

Educational challenges were also a prominent topic, with participants noting a shift in focus from education to patient care in residency programs. An emergency medicine specialist (P3) observed, “In these demanding shifts, patient care takes precedence, and education is sacrificed. This leads to a lack of interest among students in various medical fields, resulting in a shortage of competent residents.” (P4) expressed, “The residency program is tough, and some teaching hospitals treat residents as mere workers, justifying their hardships.”

Issues related to the increasing number of medical students were identified by some participants, such as participant (P11), who highlighted the crucial capacity concern, stating, “The discussion of increasing capacity is crucial. In the past, we used to admit only a few residents, but now we admit many, resulting in insufficient resources and time for proper training.”

Teaching methods were also discussed as a challenge, with participant (P4) noting, “There has been a heavy reliance on technology in education. Previously, senior professors used to conduct thorough patient examinations, but now they delegate basic tasks to assistants.” In this context, participant (P12) expressed, “In my opinion, the current educational program provided by the faculty is limited to morning reports that are held for half an hour or 45 minutes in the morning. These morning reports often lack a substantial educational aspect.” A stakeholder (P16) emphasized the importance of well-trained educators who understand the residents’ expectations and adhere to teaching principles, including their behavior, speech, and interactions.

In the third thematic category, Work-Related Challenges (Welfare Concerns, High Workload, Low Income, Communication Factors, Role and Identity, Future Career Challenges, Low Tariffs Rates):

Participants highlighted various challenges within this theme, with one of the prominent topics being welfare concerns. An outspoken individual (P6) emphasized, “We neither provide them with sufficient salaries nor offer minimum welfare facilities. We cannot even provide married housing facilities, and in these initial matters, we have not provided the necessary support, and we cannot do it.” Echoing this sentiment, another participant (P3) expressed, “On the one hand, they don’t have sufficient salaries, and on the other hand, they receive some educational assistance, but they don’t have proper insurance.” A hospital manager (P4) added, “These residents neither have insurance nor do we account for their seniority, and this is a problem.” Concurring with these concerns, a contributor (P18) stressed, “Residents should not have financial concerns. We had residents, and I called their attention to it; their salaries had increased significantly. I checked, and they hadn’t gone home for 26 nights. I asked, ‘So, when are you studying? From morning till night, you’re on duty.’ And they replied, ‘Who is going to provide a home for me?‘”.

High workload emerged as another significant challenge in residency, with participants noting that residents bear a substantial responsibility for patient care and service delivery in teaching hospitals. Participant (P1) emphasized, “Residents play a significant role in managing government hospitals. In other words, government hospitals are primarily managed by residents.” Another participant (P6) lamented, “Unfortunately, the treatment load in teaching hospitals is placed on the shoulders of residents entirely. All the treatment load, while there is a need for a ceiling in education, not only is ineffective in education but also can be destructive.” (P4) highlighted, “Long shifts are really one of the problems they have always had. 36-hour, 48-hour, and even 72-hour shifts. After all, we are all human beings, and we can make mistakes, and errors can certainly happen.”

Income challenges were identified as one of the primary concerns during the residency period. Participant (P6) remarked, “On the one hand, residents’ livelihoods are under pressure. At the global level, when someone becomes a resident, they have a minimum income, not to say that it is sufficient, but they have a minimum income that can sustain their student life, and the livelihood concern should be minimized.” A hospital director (P12) highlighted economic challenges, stating, “…from a certain point, especially since 2017, especially the little bird’s tax scheme they implemented in 2016, and as a result of that, the economic conditions of the country changed in a way that the real and monetary value emerged, and alongside that, medical tariffs did not increase at all. In 2017, surgery was 9 thousand Tomans, and after about 5 years when I came out of that structure, it had become 13 thousand and a little, compare its growth rate with the inflation rate of this country. This is an issue, the insurance companies’ failure to pay, horrible delays of 7 or 8 months for specialists, these have taken away the motivation from all residents.” A university vice-dean (P14) concurred, stating, “The most important problem in residency is this, of course, everywhere we have become accustomed to cheap labor without preserving respect.”

One of the concerns raised by the interviewees as challenges during the residency period is the communication and behavioral challenge with residents during their training. In this regard, one of the participants (P6) stated, “There are some psychological pressures in the departments, unfortunately, the educational atmosphere, they call it militarism, but it’s not like that in practice, the insults that are directed at junior assistants are a bit unjust in this space, not a beautifying environment for education.” An attendee (P8) mentioned, “I was an intern at a hospital; a first-year resident had no right to sleep, and there were three beds in that room with three interns and one resident, who took two beds and no intern could sleep there.” A specialist (P10) expressed, “Unfortunately, some have personality issues. Some do not talk properly with residents and insult or make nasty remarks everywhere.”

Another extensively discussed topic among attendees was the identity and role characteristics of residents. In this context, participant (P3) highlighted, “The motivation of students has significantly decreased, and there is no longer just a desire to get through the period; they are looking to emigrate, and others are seeking more attractive fields.” Echoing this sentiment, another interviewee (P4) observed, “In general, I can say that there has been a wave of disillusionment among resident individuals and young professionals today, which has profoundly impacted this profession.”

A significant internal factor affecting both career prospects and hospital payment capabilities is the service tariffs. Participant (P1) explained, “See, three or four events have happened simultaneously, or better say four events. First, the tariffs did not grow in line with inflation after 2014… Second, the delayed payments of insurance companies, which are a year behind. Third, the gradual increase in the insurance premium, which, as it approaches 60 million Tomans, decreases the amount between. It was supposed to reach 60 million, but now we did something that starts from 10 or 20 million to reduce, and this is very bad, and fourth, the additional graduated tax.” Another participant (P18) emphasized, “See, the tariffs are not good; for example, for a Cesarean section with an anesthetist, they deduct costs, which can be as low as 100,000 Tomans, meaning what? Calculate the cost of surgery. Putting a digit for appendectomy that the Council of Ministers approved this year is 58,000 Tomans; how much does a surgeon get? For an appendectomy operation, it’s less than 300,000 Tomans. You can get less for tube opening.”

The fourth category of challenges during the residency period comprises consequential challenges, arising from dissatisfaction or the impact of other challenges.

Participants identified these as significant issues during their residency. A hospital director (P12) shared, “In my third year of residency, I experienced severe depression with all the price increases, and I felt like I had taken a step back in the four years of studying and entering residency, and I made a mistake.” Another resident (P15) expressed, “The level of education in hospitals needs improvement; it’s a flawed cycle where they don’t pay the professor, or they force him to teach, and he has no motivation for teaching. The professor doesn’t motivate the resident, and the resident transfers this lack of motivation to the intern; it’s a flawed cycle, and all the problems are financial.” (P3) reiterated, “The motivation of students has decreased significantly, and there is no longer just a desire to get through the period; they are looking to emigrate, and others are seeking more attractive fields.”

**Fig. 1:**
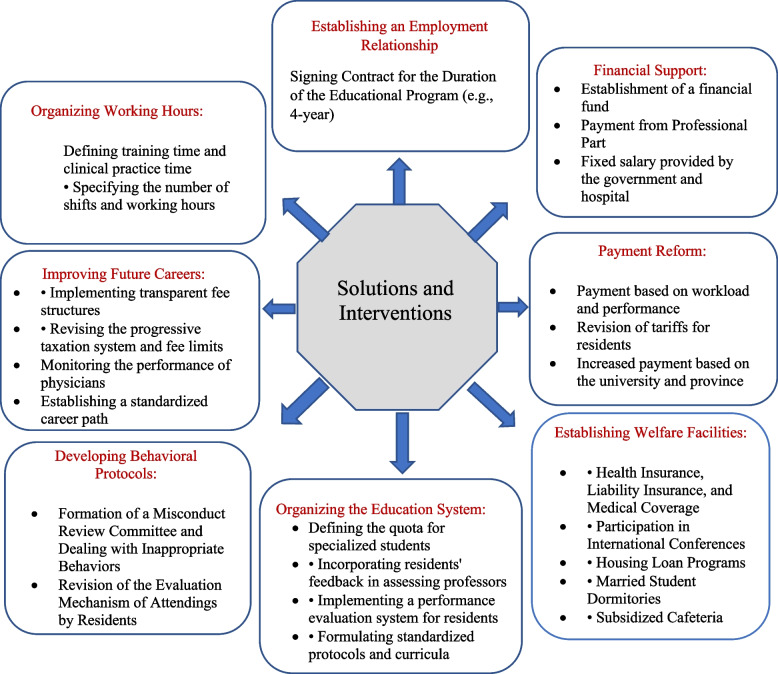
Interventions and Solutions of medical residency

In the Fig. 1, the proposed interventions and solutions, as identified in the interviews and achieving the highest level of acceptance among experts in the two Delphi rounds, are depicted.

The interviewees presented 8 main themes with 52 interventions as solutions to improve the residency period. For Delphi stage, the initial questionnaire design was formatted as a 5-mode Likert questionnaire, where individuals privately provided their judgment and vote on each option, spanning from “completely disagree” to “completely agree.” Before disseminating this questionnaire, two experts reviewed the wording of the interventions. Previous design’s proposed interventions were distributed to Delphi participants via email and, in some cases, paper, with a request for feedback. Participants in this stage could suggest deletions, additions, or modifications to the proposed plan by the providers. The culmination of judgments and votes determined the final decision. Specifically, at this stage, the criterion for acceptance was that 75% of comments had to be agreeable. Assemblies that garnered 75% or more of the votes in favor were immediately considered valid intervention. Scores falling between 50 and 70 underwent re-evaluation in a second round, with those receiving less than 50% agreement being eliminated. After two Delphi rounds involving 17 of the participants, In the first round, 3 interventions were removed, and 4 interventions that scored between 50 and 70 were reused, but they were also not accepted, these interventions were reduced to 45, with the most important interventions presented in Fig. 1.

## Discussion

Based on the findings of this qualitative study, as per the insights from experts, a proposed solution to address the challenges faced by residents is to regard their training period as a formal profession or job. The consensus is that residents play a pivotal role in delivering healthcare services within hospitals, making a significant contribution to hospital revenue. The transition to a recognized profession is seen as a potential solution to address prevalent financial and welfare-related issues, including concerns about insurance. Moreover, recognizing residency as a profession could serve as a motivating factor for residents to deliver optimal services. The authority vested in hospital boards, coupled with specific income sources within hospitals, could facilitate the transition. However, it is crucial to acknowledge and address challenges and obstacles that may impede the successful implementation of this transformative idea. The results of the residency-to-profession transformation plan, informed by the perspectives of specialists, stakeholders, and the practical experiences of other countries, indicate that such a shift is both feasible and necessary, aligning with the societal demand for these medical professionals.

Lankarani and colleagues [25], in their study, emphasize the significance of external factors, particularly structural challenges, encompassing economic difficulties, issues related to medical graduates, and social inequalities in medical education programs. Considering the numerous challenges faced by residents during their postgraduate education, which can lead to emotional distress, addressing these issues becomes imperative [26]. These challenges encompass heavy workloads, sleep deprivation, patient and family complaints, inadequate knowledge, poor educational environments, intense peer competition, unclear career paths, and social, cultural, or financial concerns. Addressing these multifaceted challenges necessitates a comprehensive plan for residents that takes into account their present and future needs.

The unique job factors and responsibilities within the medical field expose practitioners to a heightened risk of significant burnout [27]. Recognizing and addressing wellness and mental health awareness among physicians in training is vital for mitigating these risks and promoting a healthier work environment within the medical profession.

residents grapple with significant financial problems, prompting many to seek additional work in clinics during night shifts or private centers, compromising their dedicated study and research time [28]. Specialized assistants, already burdened by physician stresses, confront higher workloads, ill-defined responsibilities, and conflicting job expectations, further intensifying their stress. Additionally, the meager income during the assistantship period contributes as a stress-inducing factor [29].

Long working hours and high workloads, involving heavy patient loads and extensive service provision by residents, stand out as another formidable challenge mentioned by interviewees. The tragic case of a girl’s death in a U.S. emergency room due to excessive workload led to a reduction in working hours and a shift towards professionalism. Several countries, including Germany, England, France, the United States, Australia, Japan, South Korea, and Singapore, have established defined working hours for residents. In Europe, the maximum is set at 48 h per week, while in the United States, Singapore, and South Korea, a maximum of 80 h per week is considered. Canada ranges from 60 to 90 h, covering payment, annual leave, night shift duties, and post-service rest periods. European member states were obligated to gradually reduce residents’ working hours to less than 48 h per week by August 1, 2009 [30]. Resident working hours have also decreased in Australia and New Zealand over the past two decades. In Europe, the main driver for change has been residency, rather than patient safety [31]. The challenge arises from healthcare systems relying heavily on physicians who are not yet specialists to deliver emergency, urgent, or repetitive services, particularly outside regular office hours. Duty hour reductions primarily impact those involved in front-door emergency and urgent care, and disciplines supporting these services.

Educational challenges and teaching methods constitute another significant challenge. Postgraduate medical education plays a vital role in both education and patient care. Factors like increasing population, overcrowded emergency departments, resource shortages, and other healthcare-related problems impact medical education [32]. Specialized medical education’s role in the educational system is critical, and improving its quality is a serious concern affected by increased professor and assistant involvement in the healthcare system.

Javadi and colleagues [33], suggest key strategies for enhancing education, including standardization, quality improvement, ethical enhancement, and human dignity preservation. Improvements in education quality led to reduced teaching staff workload, increased assistant motivation, extended training time, and enhanced supervision over teaching staff and assistants’ work.

The uncertainty surrounding future careers is a contributing factor to residents’ lack of motivation. Questions about post-graduation placements and job security, coupled with inadequate income in challenging environments, dampen enthusiasm [34].

Communication-related challenges, encompassing inadequate communication with residents, disproportionate encounters, military-style interactions, and the emergence of unhealthy behaviors, pose further hurdles. Unhealthy behaviors, akin to organizational bullying, have a global impact on workplace environments [35], contributing to distress, discomfort, and physical or psychological harm. In countries with low to moderate income, personal factors, personality traits, career-related aspects, educational determinants, and interpersonal influences significantly influence specialization choices [36]. The study suggests that, especially for men residing in deprived areas, there’s a higher likelihood of staying in such areas after specialization.

The medical profession is at a critical juncture, with rising suicide rates among doctors highlighting occupational burnout [3]. The extensive effects of burnout impact physicians’ health, well-being, and patient health outcomes [37, 38]. Addressing these multifaceted challenges is imperative for the well-being of residents and the overall healthcare system.

### Study limitation

The study’s limitation lies in the inability to access certain experts for interviews and during the Delphi phase.

### Supplementary Information

Supplementary Material 1

## Data Availability

The data that support the findings of this study are not openly available due to data writing in Farsi language and are available from the corresponding author upon request.
