# Supplementary material for: Challenges and solutions of medical residency: the example of Iran
Source: BMC Health Serv Res. 2024 Jul 27;24:854. doi: 10.1186/s12913-024-11263-x (PMC11282808; doi:10.1186/s12913-024-11263-x)
Supplement: Supplementary file 1 — Supplementary Material 1 [file 12913_2024_11263_MOESM1_ESM.docx]

**Interview guide**

This interview was conducted exclusively for the research project aimed at "Conversion of Medical Residency to Job" by the Human Resources Research Center of the Ministry of Health, and this guide was tailored specifically for this project. The guide was developed through a review of relevant articles and consultations with a select group of experts. Initially, three interviews were conducted, followed by minor adjustments to the questions and their sequence.

- How do you analyze the medical residency period in Iran from your perspective?
  - Do you consider the residency course to be a job or a training program? Why?
  - How do you assess the hours, workload, and overall volume of work during the residency period?
- In teaching hospitals, is a significant volume of services provided by residents? Why?
  - Over the past 20 years, the residency period has been both exhausting and demanding. What factors have contributed to residents now experiencing job burnout, depression, and despair?
  - How do you evaluate the payment system for residents?
  - What is the nature of the relationship between professor and residents in Iran?
- What, in your view, are the other challenges of the residency period?
- What interventions have been implemented thus far to enhance the residency period, and which ones were deemed appropriate or inappropriate?
- What strategies do you propose for improving the residency period?
  - In your opinion, what actions should be taken if we intend to transition the medical residency into a job?
